# Supplementary material for: Tau tubulin kinase 1 and 2 regulate ciliogenesis and human pluripotent stem cells–derived neural rosettes
Source: Sci Rep. 2023 Aug 9;13:12884. doi: 10.1038/s41598-023-39887-9 (PMC10412607; doi:10.1038/s41598-023-39887-9)
Supplement: Supplementary file 1 — Supplementary Information. [file 41598_2023_39887_MOESM1_ESM.pdf]

Supplementary Information

Title: Tau tubulin kinase 1 and 2 regulate ciliogenesis and human pluripotent stem cells–derived neural rosettes

Authors: Lucia Binó[1] and Lukáš Čajánek[1,2, *]

Affiliations:

1: Laboratory of Cilia and Centrosome Biology, Department of Histology and Embryology, Faculty of Medicine, Masaryk University, Kamenice 3, 62500 Brno, Czech Republic

2: Section of Animal Physiology and Immunology, Department of Experimental Biology, Faculty of Science, Masaryk University, Brno, Kamenice 5, 62500 Brno, Czech Republic

* Corresponding author: cajanek@med.muni.cz

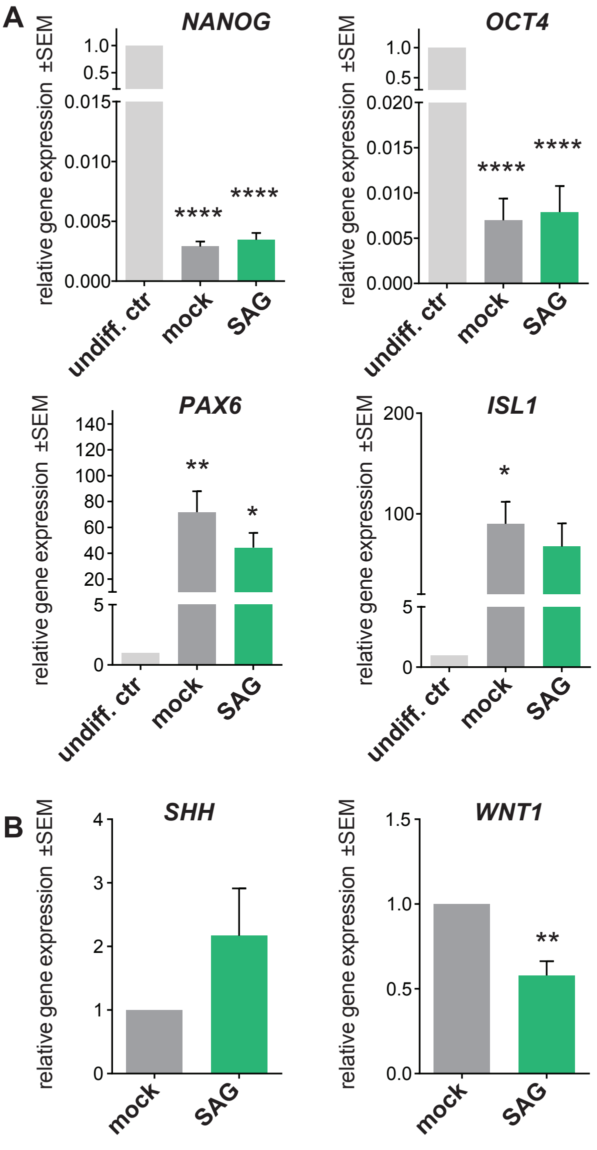

Supplementary Figure 1.
A) mRNA expression (qRT-PCR) of selected markers in mock- and SAG-treated CCTL14 rosettes on D9; n=5, one-way ANOVA with Tukey's multiple comparisons test. **B)** mRNA expression (qRT-PCR) of selected markers in mock- and SAG-treated CCTL14 rosettes on D20; n=2, t-test.

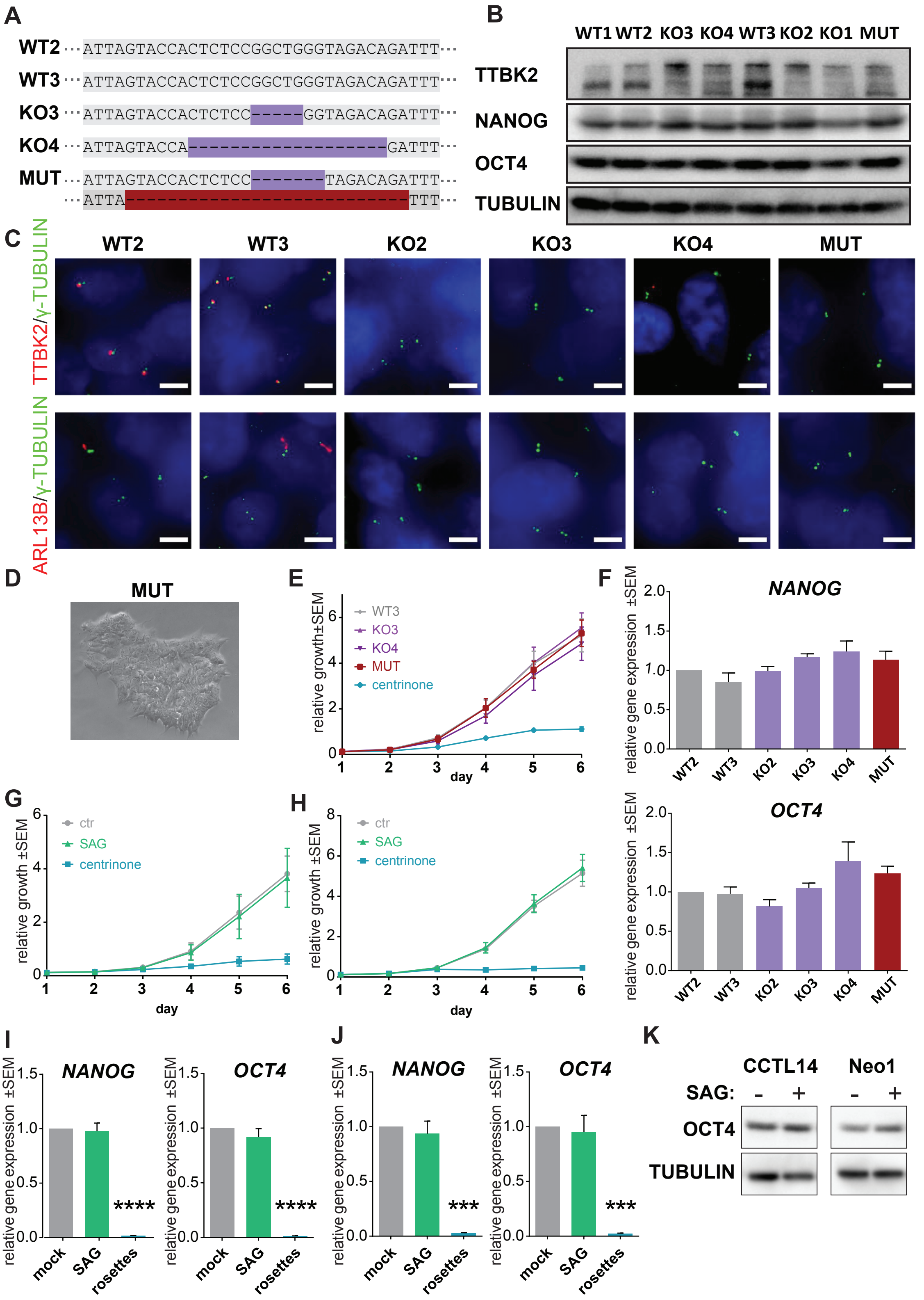

Supplementary Figure 2.

A) Schematic of the TTBK2 exon 4 sequence detail in WT and TTBK2 KO/MUT cell lines, purple=insertion/deletion, dark red=in frame deletion (mutant). **B)** Representative images of western blot detection of TTBK2, NANOG and OCT4 protein expression in undifferentiated WT and TTBK2 KO/MUT lines; α-TUBULIN was used as a loading control.. Uncropped blots shown in Suppl. Fig. 5. **C)** Representative images of IF detection of TTBK2 (top) and primary cilia (bottom; visualized by ARL13B staining) in undifferentiated WT and TTBK2 KO/MUT lines, γ-TUBULIN staining was used to detect centrioles; scalebar=5μm. **D)** Representative image of colony morphology of undifferentiated cells in TTBK2 MUT line. **E)** Relative growth comparison of indicated undifferentiated WT and TTBK2 KO/MUT lines assessed by crystal violet absorption measurement, centrinone treatment previously shown to impair the proliferation capacity was used as a control; n=3. **F)** mRNA expression (qRT-PCR) of pluripotency markers NANOG and OCT4 in indicated undifferentiated WT and TTBK2 KO/MUT lines; n=4, one-way ANOVA with Holm-Sidak's multiple comparisons test. **G)** Relative growth comparison of mock- and SAG-treated WT CCTL14 line assessed by crystal violet absorption measurement, centrinone treatment previously shown to impair the proliferation capacity was used as a control; n=3 **H)** Relative growth comparison of mock- and SAG-treated WT Neo1 line assessed by crystal violet absorption measurement, centrinone treatment previously shown to impair the proliferation capacity was used as a control; n=3. **I)** mRNA expression (qRT-PCR) of pluripotency markers NANOG and OCT4 in mock- and SAG-treated (48h) WT undifferentiated CCTL14 line, CCTL14 differentiated into neural rosettes was included for reference; n=3, one-way ANOVA with Tukey's multiple comparisons test **J)** mRNA expression (qRT-PCR) of pluripotency markers NANOG and OCT4 in mock- and SAG-treated (48h) WT undifferentiated Neo1 line, CCTL14 differentiated into neural rosettes was included for reference; n=3, one-way ANOVA with Tukey's multiple comparisons test. **K)** Representative images of western blot detection of pluripotency marker OCT4 in mock- and SAG-treated (48h) WT undifferentiated CCTL14 and Neo1; α-TUBULIN was used as a loading control. Uncropped blots shown in Suppl. Fig. 6.

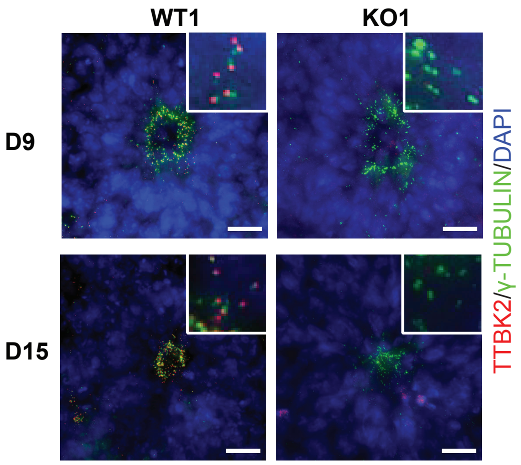

Supplementary Figure 3.
Representative images of IF detection of TTBK2 presence in WT1- and TTBK2 KO1-derived neural rosettes on D9 (top) and D15 (bottom), γ-TUBULIN was used to detect centrioles; scalebar=20µm.

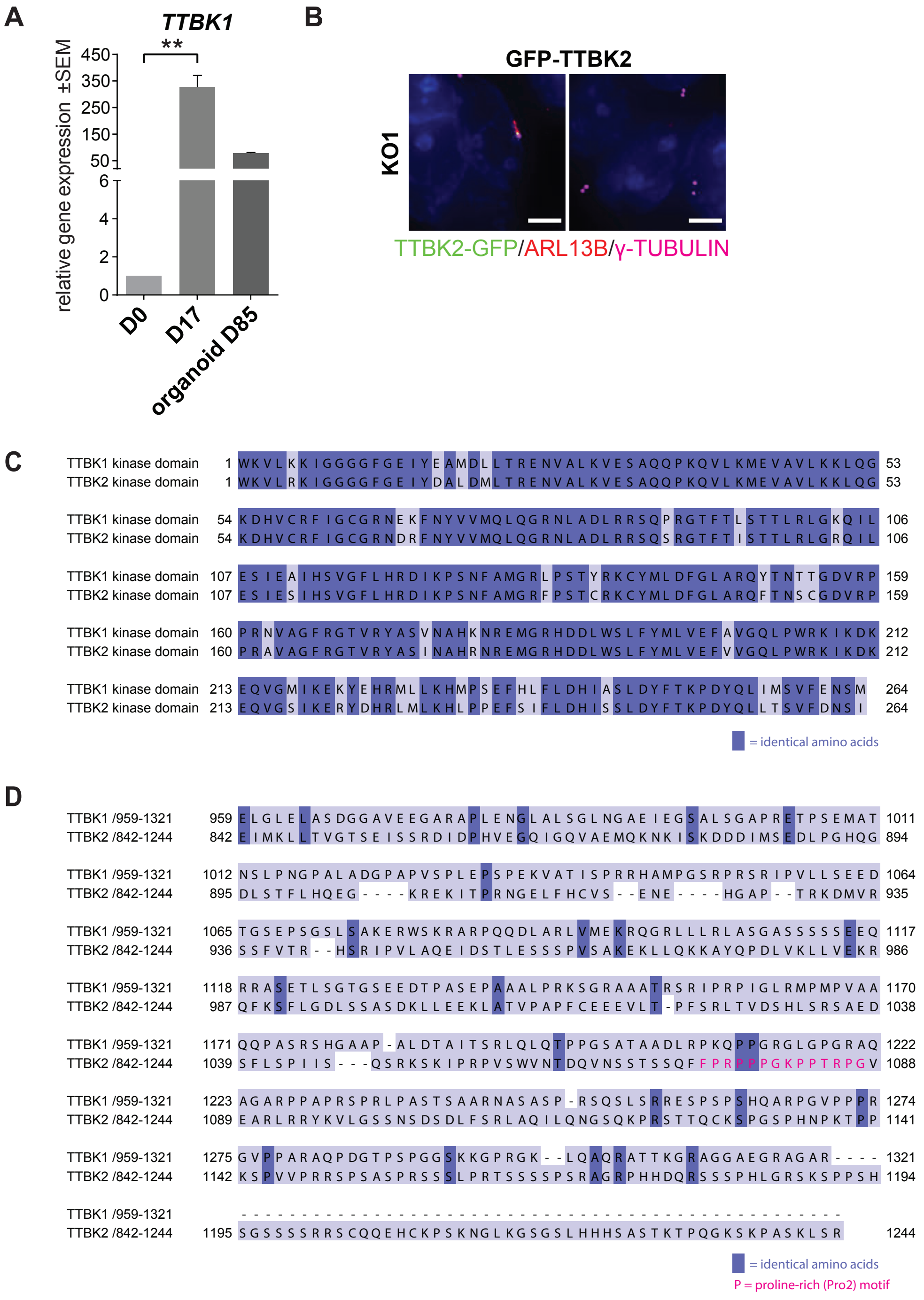

Supplementary Figure 4.

A) mRNA expression (qRT-PCR) of TTBK1 in undifferentiated parental cell line i3N compared to differentiated neurons on D17 and organoid on D85; n=2, one-way ANOVA with Holm-Sidak's multiple comparisons test. **B)** Representative images of IF detection of primary cilia (visualized by ARL13B staining) in TTBK2 KO1 cells transfected with GFP-TTBK2 (left) or not transfected (right), γ-TUBULIN was used to detect centrioles, scalebar=5µm. **C)** N-terminal TTBK1 and TTBK2 kinase domains alignment, identical amino acids are shown in dark blue. **D)** C-terminal CEP164-binding region in TTBK2 (amino acids 842-1244) aligned to TTBK1, identical amino acids are shown in dark blue, Proline-rich motif necessary for CEP164 binding is highlighted in magenta.

Supplementary Figure 5 – related to Suppl. Fig. 2B

Uncropped blot (NANOG)

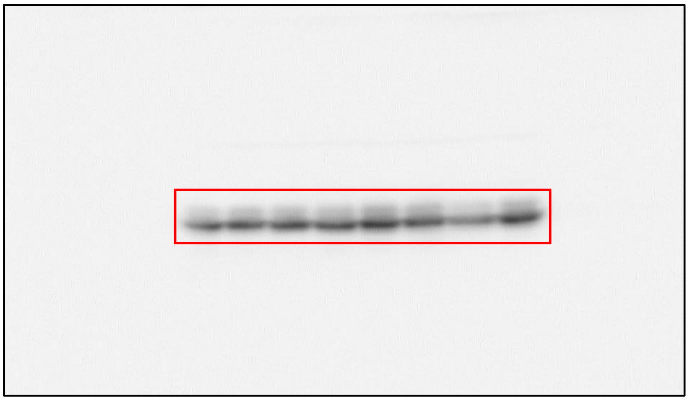

kDa membrane overlay

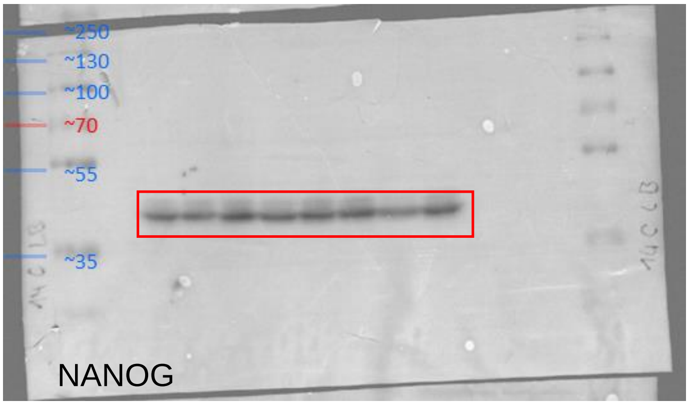

Uncropped blot
(TTBK2- upper part, α-tubulin- lower part)

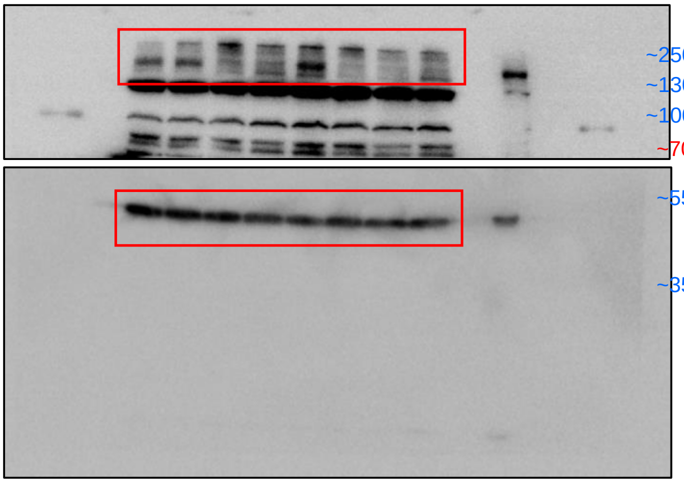

membrane overlay
(TTBK2-short exposure, tubulin-long exposure)

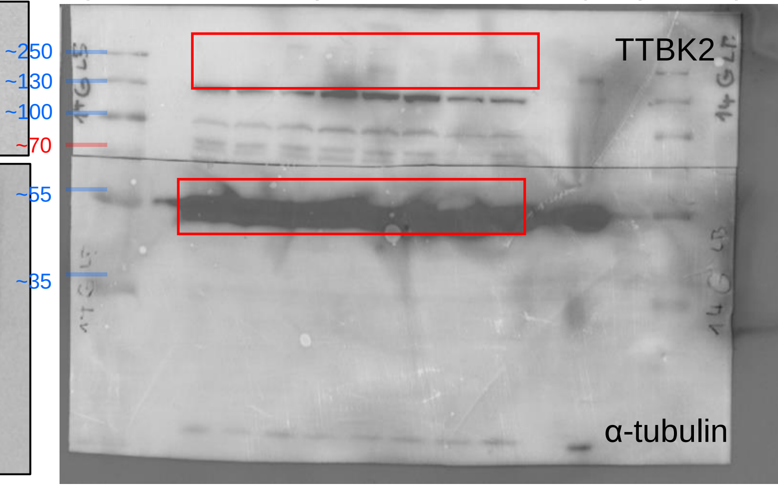

Uncropped blot (OCT4)

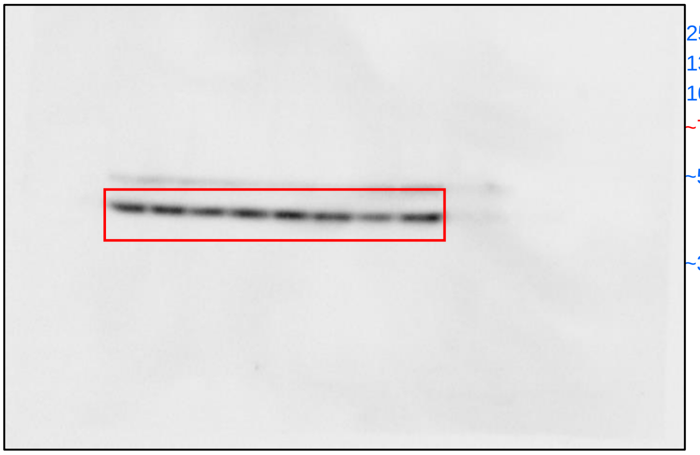

membrane overlay

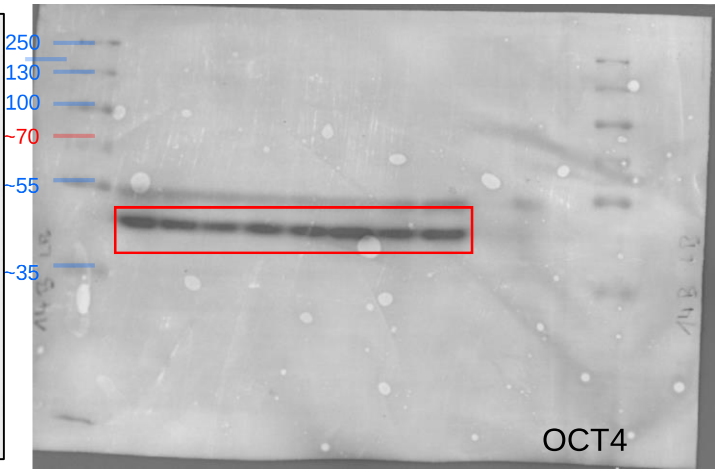

Supplementary Figure 6 – related to Suppl. Fig. 2K

Uncropped blot (OCT4)

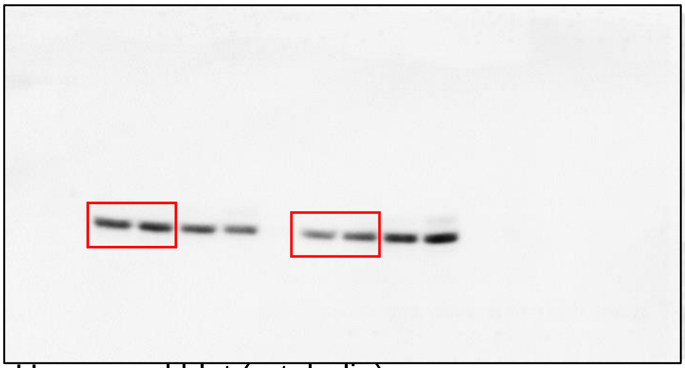

membrane overlay

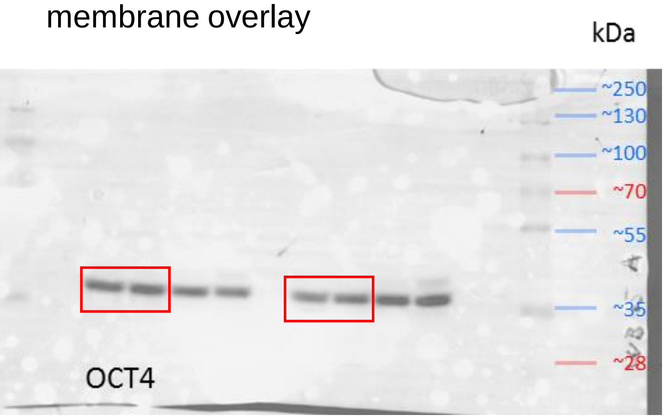

Uncropped blot (α-tubulin)

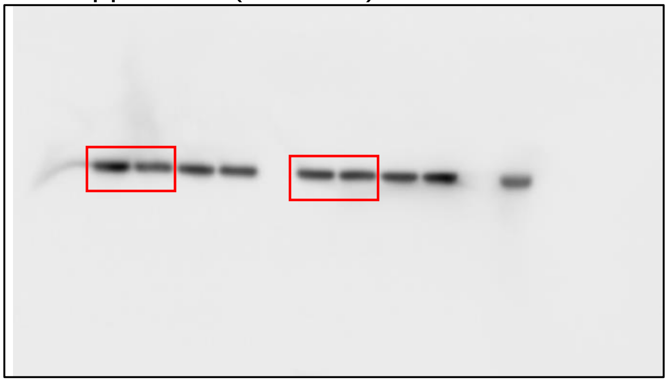

membrane overlay (long exposure blot)

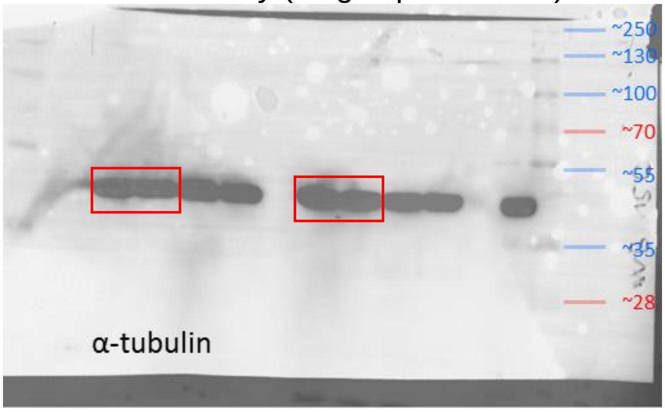

Gene	**Forward primer (5'->3')**	**Reverse primer (5'->3')**
GAPDH	AGCCACATCGCTCAGACAC	GCCCAATACGACCAAATCC
GLI1	GTGCACCACATCAACAGCGA	GGTGCGTCTTCAGGTTTTCG
PTCH1	CTTCATGGCCGCGTTAATCC	CTGACGCAGGGGCTTGTAAA
NANOG	TCTCCAACATCCTGAACCTCA	TTGCTATTCTTCGGCCAGTT
OCT4	CTTTGAGGCTCTGCAGCTTAG	CTGCTTTGCATATCTCCTGAAG
GATA6	CCATGACTCCAACTTCCACC	ACGGAGGACGTGACTTCGGC
PAX6	CGGAAGCTGCAAAGAAATAGAAC	AACTCTTTCTCCAGGGCCTCAA
ISL1	TGCTTTTCAGCAACTGGTCAAT	AGGACTGGCTACCATGCTGT
SHH	AGCGGAAGGTATGAAGGGAAG	TCAACTTGTCCTTACACCTCTGAGT
WNT1	CAAGATCGTCAACCGAGGCTGT	GCCGAAGTCAATGTTGTCGC
TTBK1	ACTACTTCACCAAGCCCGAC	GTCACATTGACCACCCCAAAC

TABLE 1: qRT-PCR Primers sequences
